# Quantification of the Pirimicarb Resistance Allele Frequency in Pooled Cotton Aphid (*Aphis gossypii* Glover) Samples by TaqMan SNP Genotyping Assay

**DOI:** 10.1371/journal.pone.0091104

**Published:** 2014-03-10

**Authors:** Yizhou Chen, Daniel R. Bogema, Idris M. Barchia, Grant A. Herron

**Affiliations:** Elizabeth Macarthur Agricultural Institute, NSW Department of Primary Industries, Menangle, New South Wales, Australia; University of New England, Australia

## Abstract

**Background:**

Pesticide resistance monitoring is a crucial part to achieving sustainable integrated pest management (IPM) in agricultural production systems. Monitoring of resistance in arthropod populations is initially performed by bioassay, a method that detects a phenotypic response to pesticides. Molecular diagnostic assays, offering speed and cost improvements, can be developed when the causative mutation for resistance has been identified. However, improvements to throughput are limited as genotyping methods cannot be accurately applied to pooled DNA. Quantifying an allele frequency from pooled DNA would allow faster and cheaper monitoring of pesticide resistance.

**Methodology/Principal Findings:**

We demonstrate a new method to quantify a resistance allele frequency (RAF) from pooled insects via TaqMan assay by using raw fluorescence data to calculate the transformed fluorescence ratio *k’* at the inflexion point based on a four parameter sigmoid curve. Our results show that *k’* is reproducible and highly correlated with RAF (r >0.99). We also demonstrate that *k’* has a non-linear relationship with RAF and that five standard points are sufficient to build a prediction model. Additionally, we identified a non-linear relationship between runs for *k’*, allowing the combination of samples across multiple runs in a single analysis.

**Conclusions/Significance:**

The transformed fluorescence ratio (*k'*) method can be used to monitor pesticide resistance in IPM and to accurately quantify allele frequency from pooled samples. We have determined that five standards (0.0, 0.2, 0.5, 0.8, and 1.0) are sufficient for accurate prediction and are statistically-equivalent to the 13 standard points used experimentally

## Introduction

Insecticide resistance has long been a problem of agriculture but has risen in prominence since the introduction of synthetic organic insecticides in the 1950’s [1]. While the use of toxins remains a fundamental method of pest control, resistance will continue to threaten sustainable agriculture. The threat remains despite the introduction of new transgenic cotton varieties and Integrated Pest Management (IPM). This is because transgenics, such as *Bt*-cotton, also rely on toxins and so expose pests to high selection for resistance. Furthermore, IPM systems favour the use of more selective compounds, thereby narrowing the range of chemicals used. One such compound is pirimicarb (Pirimor), an insecticide that is highly effective at killing aphids but not the desirable and beneficial predatory species associated with aphids [2].

Traditional monitoring of pesticide resistance in arthropods is performed by bioassay in which insects are exposed to insecticide and mortality is recorded at specific post-exposure interval(s) [3]. Resistance levels are determined from dose-response mortality data and expressed as LC_50_ values, which are an estimate of the lethal concentration required to cause 50% mortality in the target population tested [3]. Additionally, resistance can be monitored via a single diagnostic or discriminating dose but these are difficult to accurately set [4] and require the generation of significant base line data which, due to tedious laboratory process, can take weeks or months to produce.

Insecticide resistance can be behaviourally- or physiologically-based with the latter involving three distinct mechanisms: target site insensitivity, enhanced detoxification and reduced pesticide penetration [5]. With the recent advance of genomics it has been possible to study many possible target resistance genes often associated with the insect nervous system.

Examples of this include the point mutation in the *GABA* receptor conferring insecticide resistance in *Drosophila melanogaster* [6] and a mutation in the acetylcholinesterase gene causing pesticide resistance in a variety of insect species [7]. In other cases resistance is caused by detoxification linked to a single nucleotide mutation [8] or even single gene duplication or deletions [9]. However, only when these molecular mechanisms are identified can rapid molecular methods be developed, allowing more effective monitoring of pesticide resistance.

The cotton or melon aphid, *Aphis gossypii* Glover is a serious pest of many crop species including cotton, pumpkin, citrus and melons [10]. This species has developed resistance to multiple insecticides including the carbamate pirimicarb (Pirimor) and some specific organophosphates that has led to chemical control failures in Australian cotton production regions [11]. The causal mechanism of pirimicarb resistance in *A. gossypii* has been identified as target site mutation in the acetylcholinesterase gene [12], [13]. A double nucleotide substitution (TCA → TT[T/C]) in *ACE1* causes the replacement of a serine with a phenylalanine (S431F) and has been confirmed to be the cause of the pirimicarb resistance seen in Australian field collections of *A. gossypii* associated with control failure [14]. In Australian cotton IPM, a PCR-RFLP assay has been used to monitor pirimicarb resistance in the field by individually genotyping 20–50 individual aphids [2]. However, individual genotyping by PCR-RFLP limits the number of sites that can be monitored as it is labour intensive and offers limited benefits over the traditional bioassay. It is critical to have cost effective methods to monitor resistance allele frequencies (RAF) in field populations to maintain successful IPM strategies.

An alternative method to individual aphid genotyping is to estimate allele frequency from pooled DNA using real-time PCR technology with allele-specific probes or allele-specific primers [15]–[18]. However these pooled DNA approaches are often designed for specific assays and, due to the complexity of non-specific binding or amplification, are not widely used.

Currently, the most widely used qPCR platform for the estimation of allele frequency from pooled DNA is the 5′ nuclease assay. It utilizes TaqMan probes that possess a minor-groove binding (MGB) molecule and a fluorescent dye attached to the 3′ and 5′ ends, respectively. The ‘gold standard’ for this technique uses two probes with different reporter dyes, allowing the detection of both alleles. Quantification of allele frequency is achieved by using the threshold cycle (C_t_) or crossing point (CP) to calculate allele ratios based on 2^−ΔCt^ [19], [20]. However, significant variation can arise if the fluorescent probes differ significantly in their binding efficiency or if amplification efficiency varies between resistant and susceptible alleles. Yu *et al* [18] have used the normalized fluorescence ratio in the exponential phase of PCR with known premixed allele ratios and generated a linear regression from which an allele ratio can be estimated. However, this method suffers as the exponential phase of PCR is selected arbitrarily.

Here we have developed a simple method to estimate allele frequency using TaqMan assays. We show that by selecting a single, standard reference point RAF can be predicted from the ratio of the two fluorescence intensities. Additionally, we demonstrate that RAF is a function of the transformed fluorescence ratio (k’) and that five standard-points are sufficient to develop the equation of prediction.

## Materials and Methods

### PCR Assay and Probe Design for S431F

The TaqMan SNP assay was designed based on the Genbank sequence (AF502802) using RealTimeDesign Software (Biosearch Technologies) with forward primer 5′-AACCAATATACTCATGGGTAGTAACTC-3′ and the reverse primer 5′-AACCGCCGCATCTGCATT-3′. A dual-labeled probe, 5'-Quasar 670- CGAAGAGGGTTACTATTCAA-3′- BHQ2 for the susceptible allele was designed based on a known susceptible *A. gossypii* sequence for a strain known as ‘Sonya’. Two dual-labeled probes were designed for previously-identified resistance alleles, probe 5′-Fam- CGAAGAGGGTTACTATTTTA-3′-BHQ1 matching the allele identified in pirimicarb-resistant strain Adam and probe 5′-Fam-CGAAGAGGGTTACTAYTTCA-3′-BHQ1 for the allele identified in pirimicarb-resistant strain Togo. All primers and probes were synthesized by Biosearch Technologies Inc (Biosearch Technologies Inc, Novato USA).

### Predefined RAF with Plasmid DNA and Pooled Cotton Aphids

Fragments, 667 bp in size and containing the S431F mutation site, were amplified from the susceptible Sonya, and resistant Adam and Togo strains and cloned into the pCR4 vector (Invitrogen, USA) using RFLP genotyping primers. Plasmid DNA concentration was then measured by a Nanodrop 2000 (Nanodrop Technologies). To create a standard curve, a series of standards (T/S) with predefined RAF of 1.0, 0.95, 0.9, 0.8, 0.7, 0.6, 0.5, 0.4, 0.3, 0.2, 0.1, 0.05 and 0.0 were constructed by mixing plasmids containing the resistant Togo and susceptible Sonya alleles. A duplicate standard series (A/S) was made by mixing plasmids containing the Adam and Sonya alleles.

In addition to plasmid standards, a series of standards was prepared using susceptible and resistant aphids. Thirteen pools of 20 aphids were prepared with RAF of 1.0, 0.95, 0.9, 0.8, 0.7, 0.6, 0.5, 0.4, 0.3, 0.2, 0.1, 0.05 and 0.0. As an example, the pool for RAF 0.95 was constructed by extracting a tube containing 19 aphids from the resistant strain and 1 aphid from the susceptible strain.

### 2011/2012 Aphis Gossypii Field Collection

Methods for the collection, transport, culture and bioassay of *A. gossypii* samples have been described previously [11], [14]. A total of 35 *A. gossypii* samples (or strains) collected from cotton producing farms across eastern Australia during the 2011/2012 season were genotyped individually by PCR-RFLP. Resistance allele frequencies were estimated by genotyping 20 individual aphids from each sample. Samples were further confirmed susceptible or resistant via bioassay using methods outlined in detail by Herron *et al* [11].

### DNA Extraction

*Aphis gossypii* DNA was extracted from pooled or individual aphids using Chelex –100 resin (BioRad, USA) as described in [14]. Briefly, individual or 200 pooled aphids were placed inside a 1.5 mL microcentrifuge tube containing 80 µL of 5% Chelex –100 resin. The sample was thoroughly homogenized with a sterile micropestle and incubated first at 56°C for 30 min, then at 100°C for 5 min. The crude DNA sample was then used for real-time PCR or PCR-RFLP or stored at −20°C for future use.

### Individual Genotyping of S431F by PCR-RFLP

RFLP genotyping of the S431F mutation has been described previously [14]. Briefly, a 667 bp fragment containing the mutation was amplified with forward primer 5′- CAAGCCATCATGGAATCAGG-3′ and reverse primer 5′-TCATCACCATGCATCACACC-3′. The PCR product was digested by restriction endonuclease *SspI* by adding 5 units of enzyme and *SspI* buffer (1×) to a completed PCR for 3 hours at 37°C. The resultant PCR-RFLP profile was visualized by agarose gel electrophoresis. The pirimicarb-susceptible allele shows a single intense band at 336 bp (digested by *SspI*), whereas the pirimicarb-resistant allele shows a single intense band at 667 bp.

### Real-time PCR with TaqMan Assay

PCRs contained 400 nmol forward primer and reverse primer, 200 nmol susceptible and resistant probe, in a 1×TaqMan Universal PCR Master Mix (Applied Biosystems, USA) comprising a total 25 µl reaction volume. Each sample was set up in triplicate and one negative control sample was included in each run. Real-time PCR was performed in an ABI7500 Real-Time PCR System (Applied Biosystems, Foster City, CA, USA) with 10 min at 95°C followed by 47 cycles of 15 s at 95°C and 1 min at 60°C.

### Data Analysis

Sigmoid 4 parameter curve fitting statistical analysis was carried out with GENSTAT release 10 software [21] using nonlinear regression and linear regression functions.

### Principle of Quantification

Real-time PCR quantification is measured as the incremental change in signal (ΔRn) that is directly proportional to the amount of amplicons produced at any cycle [18], [22] and is defined as follows:
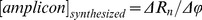
(1)Where *Δφ* represents the difference between the specific fluorescence of the free fluorophore and the specific fluorescence of the probe-bound fluorophore.

The synthesized amplicon is determined by the initial template number copy (*N_0_*), the number of cycles (*n*) and the amplification efficiency (*E*).
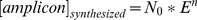
(2)


Combining equation 1 with 2 yields:
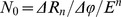
(3)


**Figure 1:**
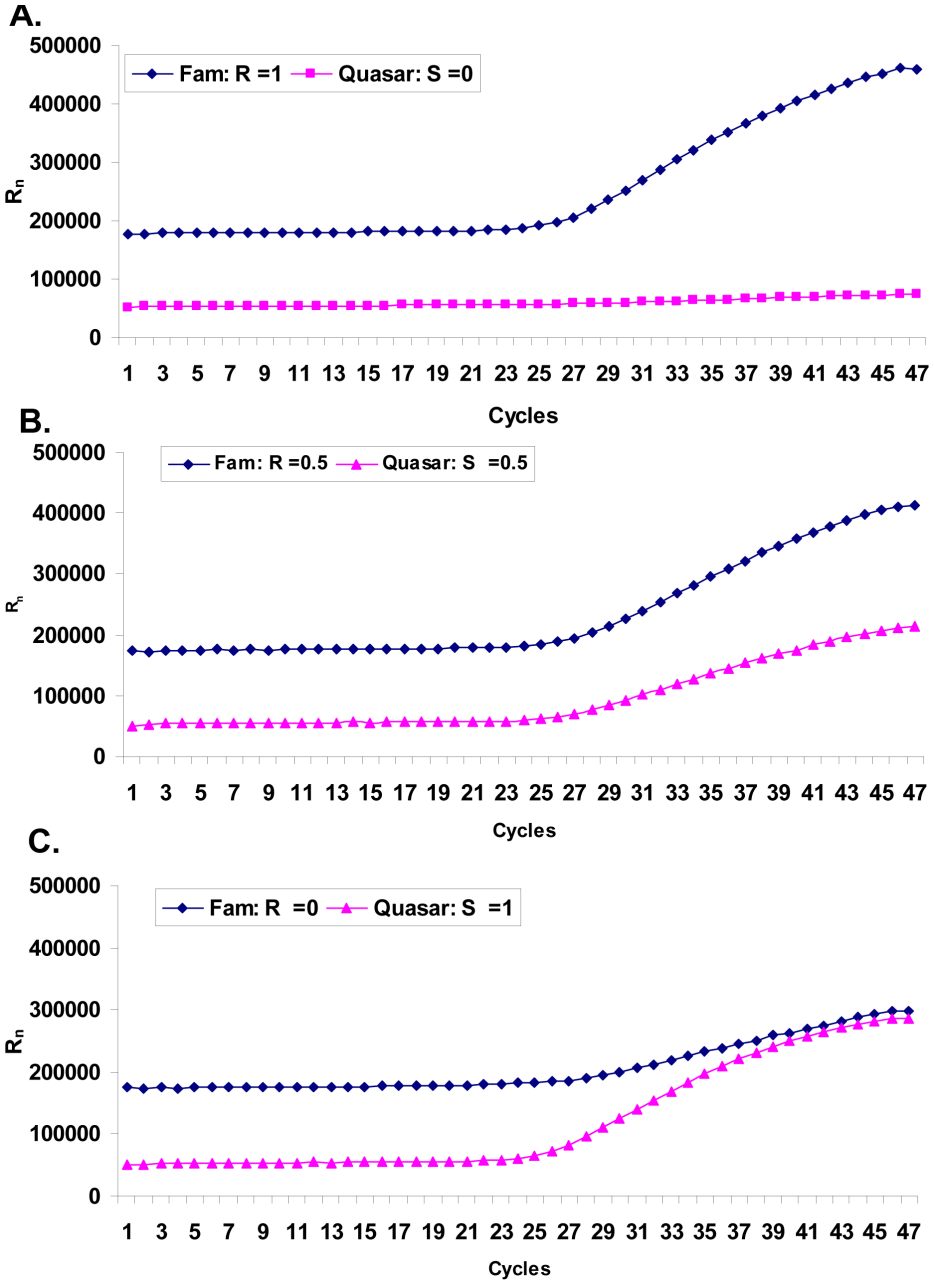
Raw fluorescence plot of TaqMan assay with two probes. Fam probe (blue) was from resistance allele and Quasar probe (red) was from susceptible allele.

Quantification of the two alleles (susceptible and resistant) was achieved with TaqMan real-time SNP assays (Figure 1). Allele R (resistance allele) and allele S (susceptible) were detected by dual-labelled probes, 5′ FAM and 3′ BHQ and 5′ Quasar 670 and 3′ BHQ, respectively.

For the resistant allele R;
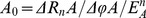
(4)where *A_0_* is the initial copy number of allele R

*ΔR_n_A* is the fluorescence intensity of Fam at cycle n.

*ΔφA* is the parameter for fluorescence Fam.

*E^n^_A_* is the compound amplification efficiency of allele R.

For the susceptible allele S:
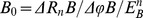
(5)Where *ΔR_n_B* is the fluorescence increment of Quasar 670 at cycle n.

The initial allele ratio:
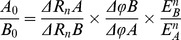
(6)


While for any given assay, the ratio of parameter *ΔφB*/*ΔφA* and *E^n^_B_*/*E^n^_A_* will be a relative constant, there will be constant relationship between *R = A_0_/B_0_* and *R’* = *ΔR_n_A*/*ΔR_n_B.*

Therefore *R* can be predicted by the ratio *R’* = *ΔR_n_A*/*ΔR_n_B.*

### Estimation of ΔR_n_

Real-time PCR is modelled via a four parametric sigmoid function [23], [24]:
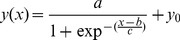
(7)Where:

*x* is cycle number,

*y(x)* is raw fluorescence of cycle x,

*y_0_* is the background fluorescence,

*a* is the maximal height of the curve (the difference between the maximal fluorescence and background fluorescence).

*b* is the first derivative maximum of the function (the inflexion point of the curve) and *c* describes the slope of the curve.

If you subtract the background fluorescence the equation above can be rewritten as:
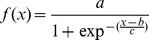
(8)Where *f(x)* is the fluorescence minus the background which is equivalent to ΔR_n._ at cycle *n*.

### Selecting a Single Point in the Exponential Phase

For allele R, with fluorescence Fam,
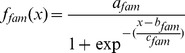
(9)Where:

*a_fam_* is the maximal height of the curve for fluorescence Fam.

*b_fam_* is the inflexion point of the curve of allele R,

*c_fam_* is the slope of the curve of allele R.

For allele S with fluorescence Quasar.
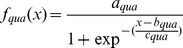
(10)Where:

*a_qua_* is the maximal height of the curve for fluorescence Quasar.

*b_qua_* is the inflexion point of the curve of allele S,

*c_qua_* is the slope of the curve of allele S.

**Figure 2:**
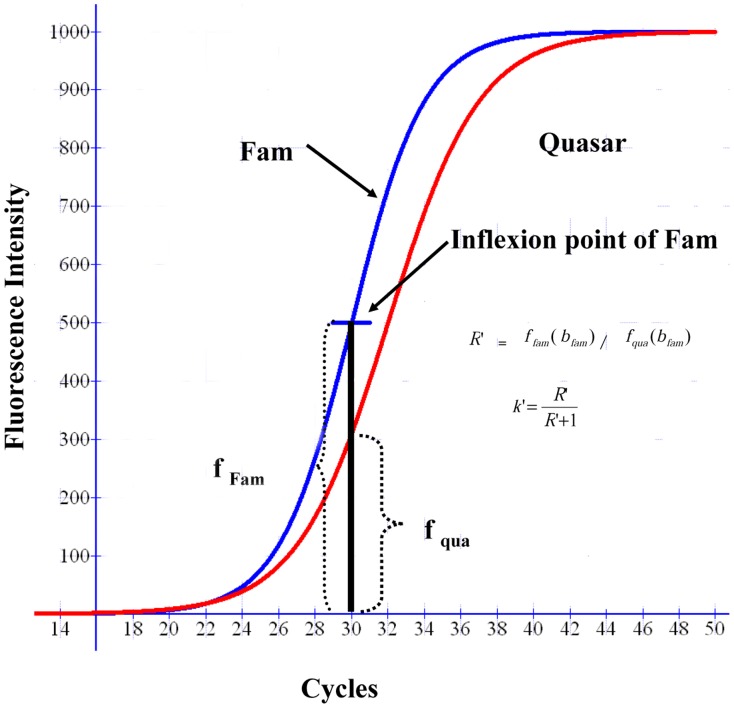
Schematic of the calculation of transformed fluorescence ratio *k'*.



Use the ratio *f_fam_(x)*/*f_qua_(x)* when one of the alleles is at its maximum speed, for example, if *b_fam_*< *b_qua_* where the Fam reaches to its maximum speed first (Figure 2).
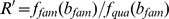



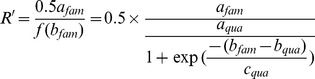





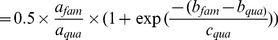
(11)


However If *b_fam_*> *b_qua_* where Quasar reaches to its maximum speed first.
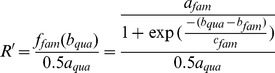





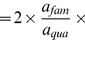
(12)


The frequency of allele can be expressed as *k.*

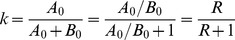
(13)


The ratio of fluorescence *R'* can be expressed as *k’.*

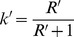
(14)


### A Two-step Sigmoid Curve Fitting for Standardized Parameters

To reduce parameter estimation bias caused by variable number of cycles in PCR plateau phase, the raw fluorescence data was fitted to a sigmoid curve twice. Fitting was performed first with all data points (in our case 47 cycles), and second fitting used data points with one slope after the inflexion point (cycle b+c) (see Table S1). An example of this calculation of k’ is demonstrated in Table S2.

## Results

### Transformed Fluorescence Ratio k'

Essentially the transformed fluorescence ratio *k'* is the transformation of the ratio of two fluorescence intensities when one of these intensities reaches its inflexion point (Figure 2). The transformed fluorescence ratio *k'* comprising 4 runs of plasmid mix and 3 runs of pooled aphids with predefined RAF is summarized in Table 1 with original data included in Table S3 and Table S9.

**Table 1:** The transformed fluorescence ratio *k’* comprising 4 runs of plasmid mix (run1-4) and 3 runs of pooled aphids (run5–7) with predefined RAF.

	Run1 T/S		Run2 A/S		Run3 A/S		Run4 T/S		Run5 MP/S		Run6 MP/S		Run7 MP/S		Inter run CV (%)
RAF	*K’*	CV (%)	*K’*	CV (%)	*K’*	CV (%)	*K’*	CV (%)	*K’*	CV (%)	*K’*	CV (%)	*K’*	CV (%)	
**100**	0.924	0.083	0.908	0.091	0.917	0.177	0.916	0.211	0.917	2.790	0.888	0.259	0.925	0.459	1.358
**95**	0.887	0.115	0.862	0.771	0.869	0.544	0.890	0.121	0.886	4.036	0.871	0.042	0.916	0.362	1.923
**90**	0.851	0.334	0.823	1.344	0.828	0.506	0.861	0.155	0.881	0.259	0.862	2.041	0.885	0.352	2.574
**80**	0.791	0.485	0.752	*	0.755	0.499	0.802	0.145	0.821	1.872	0.826	0.263	0.849	0.639	4.045
**70**	0.710	1.095	0.683	2.293	0.679	2.077	0.738	1.666	0.730	0.113	0.757	4.986	0.819	0.440	6.487
**60**	0.654	1.490	0.622	2.788	0.621	1.254	0.672	0.440	0.721	1.014	0.738	0.492	0.778	1.165	8.421
**50**	0.588	0.442	0.564	3.107	0.559	1.953	0.615	0.764	0.665	0.855	0.687	3.880	0.705	0.399	9.271
**40**	0.531	1.410	0.498	0.874	0.491	1.177	0.554	2.422	0.632	0.697	0.632	1.940	0.651	1.817	11.404
**30**	0.444	0.146	0.395	1.279	0.386	3.190	0.496	1.287	0.551	1.623	0.564	1.068	0.528	1.210	14.763
**20**	0.392	1.547	0.359	1.052	0.347	2.974	0.444	2.894	0.453	0.419	0.446	2.304	0.508	0.051	12.900
**10**	0.321	2.345	0.308	1.258	0.295	3.603	0.382	1.324	0.424	1.176	0.421	1.422	0.383	0.948	14.500
**5**	0.286	2.290	0.280	*	0.272	8.648	0.310	5.008	0.262	0.187	0.254	4.973	0.281	7.650	8.021
**0**	0.255	3.547	0.246	9.724	0.233	2.572	0.248	2.798	0.184	0.145	0.165	3.689	0.161	10.051	18.964
**Intra run** **CV (%)**		1.179		2.235		2.244		1.479		1.168		2.105		1.965	

The transformed fluorescence ratio k' is the transformation of the ratio of two fluorescence intensities when one fluorescence reaches its inflexion point.

**RAF**: Predefined Resistance allele frequency (RAF) expressed as percentage.

*K’*: Average of transformed fluorescence ratio (equation 14) from 3 replicates.

Inter run CV (%): the average coefficient of variation for a standard among 7 runs.

Intra run CV (%): the average coefficient of variation for 13 standards within a run.

*: No replicate.

The transformed fluorescence ratio *k'* is highly consistent between triplicates from each standard. The average coefficient of variation within runs (intra-run) is between 1–2%. However the variation of *k'* between runs (inter-run) for the same standard mixes is more variable and range from 1–18%. The value of *k'* within runs for each predefined standard, follow the trends of the initial RAF. As RAF becomes higher, k' also becomes higher.

### Testing the Relationship between RAF and *k'*

Using both plasmid and aphid standards, we first tested the relationship between RAF and *k'* by linear regression (as equation 6 predicts a linear relationship between these variables). In four runs using purified plasmids, a strong linear relationship was demonstrated with a high coefficient of determination (R^2^>0.99). However, the linear model did not fit as well for standards made from Chelex-extracted aphids, where the three runs produced a coefficient of determination for aphid standards ranging from 0.93 to 0.95 (also see Table S4).

In addition to linear regression, we attempted a non-linear, 4 parameter sigmoid curve fitting model. Using this non-linear model, we found that the relationship between RAF and *k'* for the three runs that used extracted aphids produced a higher coefficient of determination (R^2^>0.98), which was comparable to the purified plasmid samples. Interestingly, the purified plasmid standards also show improved coefficient of determinations using this sigmoid curve fitting model (Table S4).

### Inter-run Correlation of *k'*

Further analyses were performed to determine if there was correlation in the values of *k'* between runs. Table 2 summarizes the inter-run coefficient of determination for *k'* by linear and non-linear (sigmoid) regression. When compared like-for-like, the four plasmid and three aphid standard runs generally demonstrated a strong linear relationship (R^2^>0.98). However, in contrast to above, the linear relationship was poorer when plasmid standard runs were compared to the standards made from Chelex-extracted aphids (R^2^ = 0.89–0.97). When standard reactions were analyzed with the non-linear model a high correlation was observed between all runs. Coefficients of determination were higher (R^2^>0.99) when plasmid and Chelex-extracted aphid runs were examined with non-linear regression and compared like-for-like. When plasmid standard runs were compared to the standards made from Chelex-extracted aphids using non-linear regression, coefficients of determination were also higher (R^2^>0.97) than those generated from linear regression analysis (Table 2).

**Table 2:** Coefficient of determination R^2^ of inter-run *k’* with linear and 4 parameter sigmoid curve fitting.

R^2^	Run2 A/S	Run3 A/S	Run4 T/S	Run5 MP/S	Run6 MP/S	Run7 MP/S
**Run1 T/S - linear**	0.9975	0.997	0.9931	0.948	0.9262	0.9364
**Run1 T/S - sigmoid**	0.9989	0.9988	0.9953	0.9747	0.9855	0.9906
**Run2 A/S – linear**		0.9996	0.9859	0.9246	0.9057	0.9184
**Run2 A/S - sigmoid**		0.9999	0.9895	0.9675	0.982	0.9922
**Run3 A/S - linear**			0.9801	0.9229	0.8869	0.9091
**Run3 A/S - sigmoid**			0.9916	0.9743	0.9805	0.9884
**Run4 T/S - linear**				0.9722	0.9557	0.9582
**Run4 T/S - sigmoid**				0.9918	0.9946	0.9968
**Run5 MP/S - linear**					0.9948	0.9822
**Run5 MP/S - sigmoid**					0.9991	0.986
**Run6 MP/S - linear**						0.9869
**Run6 MP/S - sigmoid**						0.9874

Run1–4 are plasmid mix and run5–7 are pooled aphids.

### The Number of Standards Required for Accurate Prediction

The sigmoid relationship allows for a reduction in the number of data-points required to build the standard curve and hence increases the number of samples that can be examined per run. We have determined that five standards (0.0, 0.2, 0.5, 0.8, and 1.0) are sufficient for accurate prediction and are statistically-equivalent to the 13 standard points used experimentally (see Table S5, Table S6 and Table S7). We have examined RAFs using a full 13-standard model and a reduced 5-standard model for both the purified plasmid (Table S6) and Chelex-extracted aphid standard runs (Table S7). If the predefined RAF standards (0.05, 0.10, 0.30, 0.40, 0.60, 0.70, 0.90 and 0.95) were treated as unknowns, the RAFs predicted using the reduced model are highly accurate for all runs (see Table S6 and Table S7). The correlation between actual RAFs and those predicted using the reduced model standard curve is very high (R^2^>0.999).

### Combined Analysis of Multiple Runs

**Figure 3:**
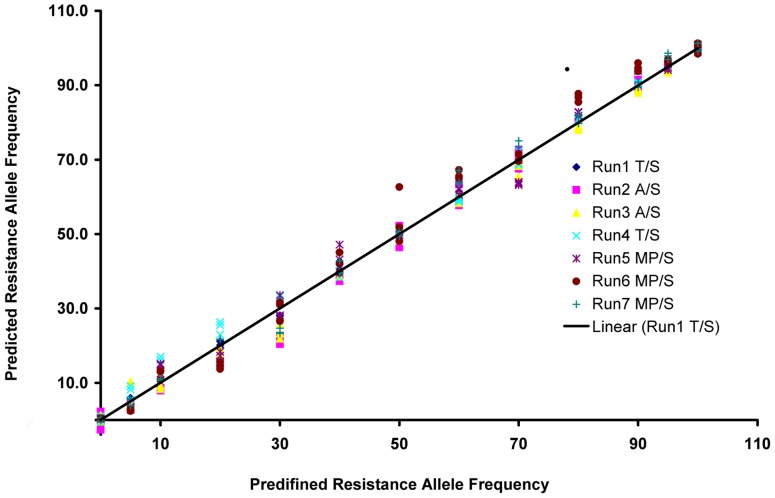
Predicted resistance allele frequency (RAF) for standards based on Run1 T/S. Prediction were based on five standards from Run1 T/S and all calculated transformed fluorescence ratio *k’* were adjusted to Run1 T/S by sigmoid function.

The sigmoid relationship between runs allows the analysis of multiple runs by normalizing all samples into a single run. The transformed fluorescence ratio *k'* for all runs was adjusted by sigmoid function using five standards shared between each run (Table S8). By normalizing to Run 1 T/S, allele frequency can now be predicted for all runs using the equation derived from this run (Table 3). The accuracy of prediction is statistically-equivalent to intra-run prediction with 13-standard-points (Figure 3).

**Table 3:** Prediction of RAF by normalizing k’ to Run1 T/S.

RAF	Run1 T/S		Run2 A/S		Run3 A/S		Run4 T/S		Run5 MP/S		Run6 MP/S		Run7 MP/S	
Standard	*k'*	RAF*	*k'* adj**	RAF*	*k'* adj	RAF*	*k'* adj	RAF*	*k'* adj	RAF*	*k'* adj	RAF*	*k'* adj	RAF*
**100**	0.924	99.9	0.927	100.3	0.926	100.2	0.921	99.5	0.925	100.0	0.918	99.0	0.923	99.7
**95**	0.887	94.5	0.885	94.2	0.885	94.2	0.895	95.6	0.881	93.5	0.883	93.9	0.907	97.4
**90**	0.851	89.2	0.849	88.9	0.849	88.8	0.864	91.1	0.873	92.5	0.865	91.2	0.854	89.6
**80**	0.791	80.2	0.783	79.0	0.784	79.1	0.798	81.3	0.789	80.0	0.798	81.2	0.794	80.7
**70**	0.71	68.1	0.717	69.1	0.713	68.6	0.724	70.2	0.668	61.8	0.686	64.5	0.746	73.5
**60**	0.654	59.7	0.657	60.1	0.659	60.4	0.646	58.4	0.657	60.1	0.659	60.4	0.684	64.2
**50**	0.588	49.7	0.599	51.3	0.599	51.3	0.579	48.3	0.590	50.0	0.593	50.4	0.585	49.2
**40**	0.531	41.1	0.531	41.1	0.531	41.2	0.510	38.0	0.553	44.4	0.531	41.2	0.522	39.7
**30**	0.444	28.0	0.422	24.8	0.424	25.0	0.449	28.8	0.471	32.1	0.467	31.5	0.409	22.7
**20**	0.392	20.2	0.384	19.0	0.383	18.8	0.399	21.3	0.391	20.1	0.381	18.6	0.394	20.5
**10**	0.321	9.7	0.328	10.6	0.327	10.5	0.345	13.3	0.371	17.0	0.366	16.3	0.322	9.8
**5**	0.286	4.5	0.297	6.0	0.302	6.8	0.292	5.3	0.284	4.2	0.289	4.9	0.284	4.2
**0**	0.255	−0.1	0.258	0.4	0.259	0.5	0.253	−0.4	0.255	0.0	0.260	0.7	0.254	−0.2
**R^2^**		0.999		0.998		0.998		0.998		0.989		0.995		0.995

RAF*: Predicted resistance allele frequency from Run1 T/S.

*k'* adj**: normalized transformed fluorescence ratio k’ based on Run1 T/S.

### Testing Pirimicarb Resistance Allele Frequency in Aphids Collected during the 2011/2012 Australian Cotton Season

To demonstrate the principle, we used qPCR to examine 35 *A. gossypii* samples collected from cotton producing farms across eastern Australia during the 2011/2012 season. Premixed DNA standards of known RAF were run simultaneously with DNAs extracted from a pool of 200 adult aphids. Table 4 lists the predicted resistance allele frequency based on *k’*. These results are consistent with resistance allele frequency obtained by individual genotyping.

**Table 4:** Field isolates of *Aphis gossypii* collected during the 2011/2012 season showing pirimicarb resistance status determined by individual PCR-RFLP.

Strain	Region	RAF by qPCR with pooled DNA	RAF by RFLP genotyping of 20 individual aphids	Bioassay
**Alch**	Darling Downs, QLD	−2.4	0	S
**And**	Fitzroy, QLD	−1.6	0	S
**Aral**	Darling Downs, QLD	−0.4	0	S
**Arra**	Darling Downs, QLD	−1.0	0	S
**Bal F3**	S. West QLD	−2.3	0	S
**Bal Vol**	S. West QLD	−2.6	0	S
**Boo Dry**	Darling Downs, QLD	−1.7	0	S
**Boo Irr**	Darling Downs, QLD	0.4	0	S
**Bor P**	S. West QLD	0.6	0	S
**Both**	Kimberley, WA	0.9	0	S
**Both B**	Kimberley, WA	102.0	100	R
**Bro Cle**	S. West QLD	−1.1	0	S
**Bro Tre**	S. West QLD	1.0	0	S
**Bud**	Darling Downs, QLD	0.9	0	S
**Bur Dry**	S. West QLD	102.0	100	R
**Car F3**	N. Inland, NSW	−1.1	0	S
**Car Vol**	N. Inland, NSW	1.0	0	S
**Carring**	N. Inland, NSW	0.9	0	S
**Cly**	S. West QLD	−1.4	0	S
**Doo 1**	S. West QLD	−1.4	0	S
**Doo 2**	S. West QLD	−0.3	0	S
**Eum**	Darling Downs, QLD	−0.6	0	S
**Fair**	Darling Downs, QLD	0.0	0	S
**Gra 148**	Fitzroy, QLD	−0.1	0	S
**Mon P**	Northern QLD	104.6	100	R
**Over**	Darling Downs, QLD	−1.2	0	S
**P Seed**	Kimberley, WA	−0.7	0	S
**Spri**	N. Inland, NSW	−0.4	0	S
**Terr**	Darling Downs, QLD	−1.3	0	S
**T Sand**	Kimberley, WA	104.1	100	R
**Walt**	Darling Downs, QLD	−1.7	0	S
**Wanh F**	Kimberley, WA	103.9	100	R
**Wise**	N. Inland, NSW	−1.3	0	S
**Wyad**	N. Inland, NSW	−1.0	0	S
**Zig**	S. West QLD	−1.7	0	S

R: Resistant to Pirimicarb.

S: Susceptible to Pirimicar.

## Discussion

The principle of quantification (Equation 6) states that the ratio of fluorescence from the two allele specific reporter dyes is a function of the initial allele ratio. Our method using the transformed fluorescence ratio at a single, standard time point is able to accurately quantify the allele frequency from pooled DNA samples and fully complies with the principle of PCR quantification. The method is less affected by background variation and so has the potential to overcome the intra-run and inter-run variation.

### Independence from Background Signal

A major contributor to observed variance in qPCR data outputs is baseline assignment and significant variation in the baseline fluorescence is often observed in replicate qPCR experiments [18], [25]. Baseline variation affects the determination of the reaction threshold yet this parameter is often set automatically by the instrument software at 10 times the standard deviation of baseline. The fluorescence baseline commonly fluctuates between wells, runs and specific instrument being used [18]. Therefore, normalizing background fluorescence often reduces the well-to well variation [25].

The transformed fluorescence ratio *k’* uses raw fluorescence data points modeled by a four-parametric sigmoid function [23], [24]. By using the transformation given in equation 8, the parameters; (*a*) the maximal height of the curve, (*b*) the first derivative maximum of the function and (*c*) the slope of the curve are less dependent on background fluorescence and the estimation of ΔRn is standardized across different wells and runs.

### Single Time Point (Inflexion Point) from Consistent Parameter Estimates

In the past decade, ‘assumption free’ quantification methods of PCR based on non-linear regression (NLR) have been developed to fit observed parameters and calculate the initial number of target molecules at cycle 0 [24], [26]–[28]. Although these models are mathematically sound and have been reported to contain less well-to-well variation, independent studies show that quantification based on these NLR methods do not outperform the conventional cycle of quantification (C_t_) method due to the increased random error of qPCR [29], [30].

One factor often unnoticed when using these models is that parameter estimates are significantly influenced by the number of cycles in the plateau phase of PCR. Sigmoid fitting methods are often not reproducible when replicate samples reach the plateau phase at slightly different cycle numbers. Our two-step sigmoid curve fitting method enables a more consistent sigmoid parameter estimate. In undertaking this method, we first fitted a sigmoid curve with all data points to obtain the proximal inflexion point (*b*) and the slope of the curve (*c*). Next the sigmoid curve was refitted with only data points from the *b*+*c* cycles. By doing that we standardized the data points so that a similar data range exists after the inflexion point for all datasets. Having an equal number of cycles after the infection point enables a more robust estimation of the parameters.

### A Single Reference Point for Fluorescence Ratio Determination

Earlier work by Oliver *et al* [31] to quantify the initial allele ratio by examining the qPCR end point fluorescence ratio is not ideal for accurate quantification due to the dramatic decrease of amplification efficiency in late PCR cycles. To more accurately predict the initial allele ratio, Yu *et al* [18] used background-normalized fluorescence from both fluorophores in exponential phase. However, the selection of the exponential cycles in this method was arbitrary, particularly when one fluorescence signal reaches the exponential phase much earlier than the other which is often the case when one or the other allele frequency is quite low. In our two-step sigmoid curve fitting method, the fluorescence ratio is measured when a fluorescence signal first reaches the inflexion point (equation 11 and 12) and allows for a standard method of identifying the exponential phase. As the inflexion point is always in the middle of the exponential phase it shows very similar kinetics between replicate samples and so has the potential to be more accurate.

### Transformed Fluorescence Ratio *k’*

The transformed fluorescence ratio *k’* (Equation 13) permits the development of a standard curve with allele frequency ranging from 0 to 1. The inclusion of a zero allele frequency is critical as a control to assess the sensitivity of the assay. In TaqMan assays, potential errors occur due to significant cross-binding of probes. Even when one allele is absent its corresponding florescence signal can still be observed due to cross binding. Including an allele frequency of 0 and 1 makes it possible to accurately estimate unknown samples with allele frequency <0.05 or <0.95.

### Non-linear Relationship between Transformed Fluorescence Ratio *k’* and RAF

Our results demonstrate a sigmoid relationship between RAF and transformed fluorescence ratio *k’*. The predicted linear relationship between the initial allele frequency and transformed fluorescence ratio *k’* can be achieved for PCR in optimal conditions. However, a non-linear model is more universal given most PCRs are performed in conditions that are not optimal, particularly when unknown PCR inhibitors are present.

The sigmoid function between RAF and transformed fluorescence ratio *k’* theoretically enables the construction of a standard curve using only 4 standard points and our results demonstrate that the prediction model obtained from 5 rather than 4 standard points was as accurate as the model base on 13 standard points. This reduction in the number of standards required for each run allows for a considerable increase in the number of wells that can be used for samples rather than standards.

### Inter-run Correlation

Additionally, we have found that there is a sigmoid relationship between the transformed fluorescence ratios *k’* across multiple runs. This enables the normalization of samples from multiple experiments into a single run if at least 4 samples are shared in each. Therefore, a single analysis can be performed for all samples across all runs.

### Practical Implementation of the Method

Our two-step sigmoid curve fitting method has the potential to be used broadly for high throughput/low cost genotyping. Although this method was developed using a TaqMan assay on an ABI 7500 real-time thermocycler, the principle can theoretically be applied to other fluorescent dye and instrument platforms (such as SYBR green). In some cases, when one allele is absent, there is no PCR amplification or irregular amplification, it is possible to manually estimate fluorescence height above the background at the approximate inflexion point of the other allele for the *k’* calculation. Alternatively, a predefined allele frequency 0.01 and 0.99 can be used as standard points.

### Diagnostic Testing the Pirimicarb Resistance Allele Frequency in Aphids Collected during the 2011/2012 Cotton Season

To examine the effectiveness of our two-step sigmoid curve fitting method we used field samples to predict the pirimicarb-RAF in 35 field isolates of *A. gossypii* and compared those estimated allele frequencies with individual genotyping of 20 aphids from each isolate. A remarkable consistency was observed between the RAF predicted by qPCR and allele frequency predicted by individual genotyping. Unfortunately, the pirimicarb-RAF observed in the 2011/2012 season where either 0% or 100%, making it difficult to statistically assess the precision of the prediction. While this data limitation could not be overcome, the method demonstrated good sensitivity when RAF is low.

This method allows for a dramatic decrease in the amount of labor required for the high-throughput monitoring of RAF in insects of agricultural importance, so aiding sustainable IPM systems. Interestingly, we found that a similar amount of time was required for an experienced worker to extract DNA from 20 aphids individually or 200 aphids combined in one tube. However, there was a great difference in the amount of time required to genotype these samples. Genotyping of 35×20 aphids individually required almost three weeks of work while genotyping of 35×200 aphids using pooled DNA, a TaqMan assay and our two-step sigmoid curve fitting method could be performed in as little as three days.

## Conclusion

We have developed a method using a TaqMan SNP assay to accurately estimate the allele frequencies from pooled DNA samples. The method uses the transformed fluorescence ratio based on a single reference point and has proven precise at predicting unknown allele frequencies. The prediction model can be built using five standard points and results can be normalized across multiple runs. The method can dramatically reduce time and labour required for insecticide resistance monitoring and has the potential for broad applications in high throughput genotyping such as genome –wide association studies, population studies even the quantitative assessment of post transplant chimeras in medicine.

## Supporting Information

Table S1 **An example of two-step four-parameter sigmoid curve fitting.**
(DOC)

Table S2 **Example of Calculation of** ***k'*** **.**
(DOC)

Table S3 **The transformed fluorescence ratio** ***k’*** **comprising 4 runs of plasmid mix (run1-4) and 3 runs of pooled aphids (run5-7) with predefined resistance allele frequency (RAF).** The transformed fluorescence ratio *k'* is the transformation of the ratio of two fluorescence intensity when one fluorescence reaches its inflexion point.
(DOC)

Table S4 **Test of the linear and non-linear (sigmoid) relationship between RAF and transformed fluorescence ratio** ***k'*** **. Run1-4 are plasmid mix and run5-7 are pooled aphids.**
(DOC)

Table S5 **Five standard points used for the reduced prediction model.**
(DOC)

Table S6 **Resistance allele frequencies (RAF) predicted from full and reduced prediction models in plasmid mix runs.**
(DOC)

Table S7 **Resistance allele frequencies (RAF) predicted from full and reduced prediction models in aphid mix runs.**
(DOC)

Table S8 **Transformed fluorescence ratio** ***k’*** **between runs can be normalized to Run1 T/S by using five standards based on sigmoid function.**
(DOC)

Table S9 **Raw fluorescence data for 7 runs.**
(CSV)
