# Supplementary material for: Quantification of the Pirimicarb Resistance Allele Frequency in Pooled Cotton Aphid (Aphis gossypii Glover) Samples by TaqMan SNP Genotyping Assay
Source: PLoS One. 2014 Mar 10;9(3):e91104. doi: 10.1371/journal.pone.0091104 (PMC3948748; doi:10.1371/journal.pone.0091104)
Supplement: Table S1 — An example of two-step four-parameter sigmoid curve fitting. (DOC) [file pone.0091104.s001.doc]

**Table S1.** An example of two-step four-parameter sigmoid curve fitting

| **Cycle number** | **Raw fluorescence (FAM)*** | **Raw fluorescence (FAM)**** |
| --- | --- | --- |
| 1 | 175938 | 175938 |
| 2 | 175181 | 175181 |
| 3 | 175254 | 175254 |
| 4 | 175581 | 175581 |
| 5 | 175708 | 175708 |
| 6 | 176668 | 176668 |
| 7 | 176401 | 176401 |
| 8 | 176573 | 176573 |
| 9 | 175926 | 175926 |
| 10 | 176494 | 176494 |
| 11 | 176768 | 176768 |
| 12 | 176272 | 176272 |
| 13 | 176918 | 176918 |
| 14 | 177557 | 177557 |
| 15 | 177501 | 177501 |
| 16 | 178681 | 178681 |
| 17 | 178763 | 178763 |
| 18 | 179271 | 179271 |
| 19 | 179174 | 179174 |
| 20 | 179925 | 179925 |
| 21 | 179918 | 179918 |
| 22 | 180583 | 180583 |
| 23 | 181493 | 181493 |
| 24 | 183653 | 183653 |
| 25 | 187203 | 187203 |
| 26 | 192940 | 192940 |
| 27 | 202261 | 202261 |
| 28 | 213759 | 213759 |
| 29 | 228409 | 228409 |
| 30 | 244968 | 244968 |
| 31 | 261954 | 261954 |
| 32 | 281126 | 281126 |
| 33 | 297253 | 297253 |
| 34 | 314020 | 314020 |
| 35 | 330092 | 330092 |
| 36 | 344746 | 344746 |
| 37 | 359066 | 359066 |
| 38 | 372197 | 372197 |
| 39 | 383812 |  |
| 40 | 395170 |  |
| 41 | 405989 |  |
| 42 | 415980 |  |
| 43 | 425375 |  |
| 44 | 435188 |  |
| 45 | 442102 |  |
| 46 | 448990 |  |
| 47 | 452060 |  |

| Y= a / (1.0 + exp(-(x-b)/c)) + y_0_ |  |  |
| --- | --- | --- |
| **Parameter** |  |  |
| a | 280038 | 216271 |
| b | 34.3 | 32.3 |
| c | 3.8 | 2.77 |
| y_0_ | 174148 | 176192 |
| R^2^ | 0.998 | 0.999 |

*: Step 1 with full data set 47 cycles)

**: Step2 with b+c data points 34.3+3.8 =38 Cycle)
