# Supplementary material for: Quantification of the Pirimicarb Resistance Allele Frequency in Pooled Cotton Aphid (Aphis gossypii Glover) Samples by TaqMan SNP Genotyping Assay
Source: PLoS One. 2014 Mar 10;9(3):e91104. doi: 10.1371/journal.pone.0091104 (PMC3948748; doi:10.1371/journal.pone.0091104)
Supplement: Table S2 — Example of Calculation of k' . (DOC) [file pone.0091104.s002.doc]

**Table S2.** Example of Calculation of k'

**A. when b_fam_ <b_qua_ using equation 11**

| **Sample** | **a_fam_** | **b_fam_** | **c_fam_** | **R'** | ***k'*** | **b_fam_-b_qua_** | **a_qua_** | **b_qua_** | **c_qua_** |
| --- | --- | --- | --- | --- | --- | --- | --- | --- | --- |
| **T/S100** | 216270 | 32.3 | 2.8 | 12.38 | **0.9253** | -6.3 | 27524 | 38.6 | 8.2 |
| **T/S100** | 237192 | 32.2 | 2.8 | 12.18 | **0.9241** | -6.4 | 31846 | 38.7 | 7.8 |
| **T/S100** | 220890 | 32.2 | 2.8 | 12.13 | **0.9238** | -6.6 | 29596 | 38.8 | 8.1 |

**B. when b_fam_ <b_qua_ using equation 12**

| **Sample** | **a_fam_** | **b_fam_** | **c_fam_** | **R'** | ***k'*** | **b_fam_-b_qua_** | **a_qua_** | **b_qua_** | **c_qua_** |
| --- | --- | --- | --- | --- | --- | --- | --- | --- | --- |
| **T/S50** | 193132 | 33.4 | 3.1 | 1.44 | **0.5908** | 0.4 | 126047 | 33.0 | 3.2 |
| **T/S50** | 195465 | 33.3 | 3.0 | 1.42 | **0.5867** | 0.4 | 129207 | 33.0 | 3.1 |
| **T/S50** | 194311 | 33.4 | 3.1 | 1.42 | **0.5860** | 0.3 | 129519 | 33.0 | 3.2 |
| **T/S0** | 115502 | 35.6 | 3.9 | 0.35 | **0.2612** | 3.6 | 186561 | 32.1 | 2.8 |
| **T/S0** | 110981 | 34.8 | 3.8 | 0.35 | **0.2583** | 3.4 | 184090 | 31.4 | 2.7 |
| **T/S0** | 106871 | 35.6 | 4.1 | 0.32 | **0.2443** | 4.1 | 177846 | 31.5 | 2.7 |

$a_{fam}$: Maximal height of the curve for fluorescence FAM, $b_{fam}$ : Inflexion point of the curve of allele R, $c_{fam}$ : Slope of the curve of allele R

$a_{qua}$ : Maximal height of the curve for fluorescence Quasar 670 , $b_{qua}$ : Inflexion point of the curve of allele S,

$c_{qua}$ : Slope of the curve of allele S, $k'$ : Transformed fluorescence ratio
