# Supplementary material for: Quantification of the Pirimicarb Resistance Allele Frequency in Pooled Cotton Aphid (Aphis gossypii Glover) Samples by TaqMan SNP Genotyping Assay
Source: PLoS One. 2014 Mar 10;9(3):e91104. doi: 10.1371/journal.pone.0091104 (PMC3948748; doi:10.1371/journal.pone.0091104)
Supplement: Table S3 — The transformed fluorescence ratio k’ comprising 4 runs of plasmid mix (run1-4) and 3 runs of pooled aphids (run5-7) with predefined resistance allele frequency (RAF). The transformed fluorescence ratio k' is the transformation of the ratio of two fluorescence intensity when one fluorescence reaches its inflexion point. (DOC) [file pone.0091104.s003.doc]

**Table S3.** The transformed fluorescence ratio $k'$ comprising 4 runs of plasmid mix (run1-4) and 3 runs of pooled aphids (run5-7) with predefined resistance allele frequency (RAF). The transformed fluorescence ratio k' is the transformation of the ratio of two fluorescence intensity when one fluorescence reaches it’s inflexion point.

| **RAF** | **k'** | | | | | | |
| --- | --- | --- | --- | --- | --- | --- | --- |
|  | **Run1 T/S** | **Run2 A/S** | **Run3 A/S** | **Run4 T/S** | **Run5 MP/S** | **Run6 MP/S** | **Run7 MP/S** |
| **100** | 0.9253 | 0.9075 | 0.9169 | 0.9183 | 0.9216 | 0.8943 | 0.9299 |
| **100** | 0.9241 | 0.9085 | 0.9187 | 0.9155 | 0.9183 | 0.8901 | 0.9241 |
| **100** | 0.9238 | 0.9091 | 0.9155 | 0.9145 | 0.9101 | 0.8802 | 0.9216 |
| **95** | 0.8885 | 0.8668 | 0.8646 | 0.8910 | 0.8912 | 0.8725 | 0.9193 |
| **95** | 0.8871 | 0.8574 | 0.8741 | 0.8898 | 0.8847 | 0.8724 | 0.9164 |
| **95** | 0.8865 |  | 0.8691 | 0.8889 | 0.8816 | 0.8695 | 0.9126 |
| **90** | 0.8533 | 0.8310 | 0.8235 | 0.8618 | 0.8810 | 0.8681 | 0.8885 |
| **90** | 0.8524 | 0.8154 | 0.8295 | 0.8611 | 0.8804 | 0.8613 | 0.8832 |
| **90** | 0.8480 |  | 0.8315 | 0.8592 | 0.8803 | 0.8576 | 0.8830 |
| **80** | 0.7909 | 0.7523 | 0.7553 | 0.8030 | 0.8224 | 0.8302 | 0.8534 |
| **80** | 0.7944 |  | 0.7507 | 0.8010 | 0.8218 | 0.8261 | 0.8512 |
| **80** | 0.7868 |  | 0.7581 | 0.8010 | 0.8174 | 0.8204 | 0.8431 |
| **70** | 0.7165 | 0.6666 | 0.6826 | 0.7522 | 0.7320 | 0.7613 | 0.8234 |
| **70** | 0.7118 | 0.6862 | 0.6904 | 0.7320 | 0.7302 | 0.7533 | 0.8172 |
| **70** | 0.7013 | 0.6975 | 0.6630 | 0.7301 | 0.7283 |  | 0.8171 |
| **60** | 0.6620 | 0.6148 | 0.6198 | 0.6754 | 0.7237 | 0.7434 | 0.7884 |
| **60** | 0.6565 | 0.6086 | 0.6300 | 0.6724 | 0.7232 | 0.7360 | 0.7739 |
| **60** | 0.6431 | 0.6412 | 0.6147 | 0.6695 | 0.7158 | 0.7339 | 0.7717 |
| **50** | 0.5908 | 0.5441 | 0.5677 | 0.6190 | 0.6719 | 0.7241 | 0.7073 |
| **50** | 0.5867 | 0.5708 | 0.5631 | 0.6169 | 0.6632 | 0.6771 | 0.7066 |
| **50** | 0.5860 | 0.5771 | 0.5469 | 0.6100 | 0.6591 | 0.6601 | 0.7021 |
| **40** | 0.5398 | 0.4925 | 0.4942 | 0.5619 | 0.6498 | 0.6461 | 0.6643 |
| **40** | 0.5274 | 0.4997 | 0.4839 | 0.5611 | 0.6291 | 0.6315 | 0.6449 |
| **40** | 0.5264 | 0.5003 | 0.4936 | 0.5383 | 0.6164 | 0.6191 | 0.6430 |
| **30** | 0.4444 | 0.3907 | 0.3837 | 0.5027 | 0.5747 | 0.5755 | 0.5356 |
| **30** | 0.4435 | 0.4007 | 0.3749 | 0.4962 | 0.5401 | 0.5720 | 0.5260 |
| **30** |  | 0.3945 | 0.3992 | 0.4899 | 0.5371 | 0.5439 | 0.5235 |
| **20** | 0.3983 | 0.3565 | 0.3472 | 0.4543 | 0.4645 | 0.4562 | 0.5087 |
| **20** | 0.3909 | 0.3618 | 0.3368 | 0.4488 | 0.4551 | 0.4445 | 0.5083 |
| **20** | 0.3863 |  | 0.3575 | 0.4298 | 0.4404 | 0.4371 | 0.5082 |
| **10** | 0.3297 | 0.3103 | 0.2858 | 0.3851 | 0.4342 | 0.4323 | 0.3866 |
| **10** | 0.3164 | 0.3048 | 0.2916 | 0.3780 | 0.4311 | 0.4297 | 0.3812 |
| **10** | 0.3169 |  | 0.3064 |  | 0.4058 | 0.4021 | 0.3797 |
| **5** | 0.2939 | 0.2796 | 0.2593 | 0.3224 | 0.2695 | 0.2591 | 0.2959 |
| **5** | 0.2835 |  | 0.2577 | 0.3152 | 0.2647 | 0.2589 | 0.2901 |
| **5** | 0.2818 |  | 0.2992 | 0.2926 | 0.2515 | 0.2429 | 0.2562 |
| **0** | 0.2612 | 0.2607 | 0.2322 | 0.2542 | 0.1865 | 0.1715 | 0.1714 |
| **0** | 0.2583 | 0.2579 | 0.2392 | 0.2500 | 0.1862 | 0.1633 | 0.1701 |
| **0** | 0.2443 | 0.2180 | 0.2272 | 0.2406 | 0.1793 | 0.1614 | 0.1427 |

**RAF**: predefined resistance allele frequency expressed as percentage

**k'**: transformed fluorescence ratio with equation 14
