# Supplementary material for: Quantification of the Pirimicarb Resistance Allele Frequency in Pooled Cotton Aphid (Aphis gossypii Glover) Samples by TaqMan SNP Genotyping Assay
Source: PLoS One. 2014 Mar 10;9(3):e91104. doi: 10.1371/journal.pone.0091104 (PMC3948748; doi:10.1371/journal.pone.0091104)
Supplement: Table S4 — Test of the linear and non-linear (sigmoid) relationship between RAF and transformed fluorescence ratio k' . Run1-4 are plasmid mix and run5-7 are pooled aphids. (DOC) [file pone.0091104.s004.doc]

**Table S4.** Test of the linear and non-linear (sigmoid) relationship between RAF and transformed fluorescence ratio $k'$. Run1-4 are plasmid mix and run5-7 are pooled aphids

|  | **Run1 T/S** | **Run2 A/S** | **Run3 A/S** | **Run4 T/S** | **Run5 MP/S** | **Run6 MP/S** | **Run7 MP/S** |
| --- | --- | --- | --- | --- | --- | --- | --- |
| **RAF** | $k'$ | $k'$ | $k'$ | $k'$ | $k'$ | $k'$ | $k'$ |
| **100** | 0.924 | 0.908 | 0.917 | 0.916 | 0.917 | 0.888 | 0.925 |
| **95** | 0.887 | 0.862 | 0.869 | 0.89 | 0.886 | 0.871 | 0.916 |
| **90** | 0.851 | 0.823 | 0.828 | 0.861 | 0.881 | 0.862 | 0.885 |
| **80** | 0.791 | 0.752 | 0.755 | 0.802 | 0.821 | 0.826 | 0.849 |
| **70** | 0.71 | 0.683 | 0.679 | 0.738 | 0.73 | 0.757 | 0.819 |
| **60** | 0.654 | 0.622 | 0.621 | 0.672 | 0.721 | 0.738 | 0.778 |
| **50** | 0.588 | 0.564 | 0.559 | 0.615 | 0.665 | 0.687 | 0.705 |
| **40** | 0.531 | 0.498 | 0.491 | 0.554 | 0.632 | 0.632 | 0.651 |
| **30** | 0.444 | 0.395 | 0.386 | 0.496 | 0.551 | 0.564 | 0.528 |
| **20** | 0.392 | 0.359 | 0.347 | 0.444 | 0.453 | 0.446 | 0.508 |
| **10** | 0.321 | 0.308 | 0.295 | 0.382 | 0.424 | 0.421 | 0.383 |
| **5** | 0.286 | 0.28 | 0.272 | 0.31 | 0.262 | 0.254 | 0.281 |
| **0** | 0.255 | 0.246 | 0.233 | 0.248 | 0.184 | 0.165 | 0.161 |

Y= a + bx

| **a** | 0.2543 | 0.2337 | 0.2197 | 0.2928 | 0.2978 | 0.2988 | 0.2992 |
| --- | --- | --- | --- | --- | --- | --- | --- |
| **b** | 0.0067 | 0.0067 | 0.0068 | 0.0063 | 0.0065 | 0.0065 | 0.0069 |
| **R^2^** | 0.9994 | 0.9965 | 0.9957 | 0.9936 | 0.9471 | 0.9266 | 0.9359 |

Y= a / (1.0 + exp(-(x-b)/c)) + y_0_

| **a** | -109.7 | -2.7 | -2.9 | -9916.8 | -122910. | -153681. | -154772. |
| --- | --- | --- | --- | --- | --- | --- | --- |
| **b** | 3304.8 | 105.0 | 111.9 | -2349.6 | -764.1 | -585.3 | -616.2 |
| **c** | -3223.3 | -94.8 | -94.1 | -277.7 | -64.3 | -48.0 | -50.8 |
| **y_0_** | 80.97 | 2.32 | 2.43 | 2.4 | 1.1 | 0.97 | 1.0 |
| **R^2^** | 0.9994 | 0.9980 | 0.9973 | 0.9957 | 0.9802 | 0.9861 | 0.9915 |
