# Supplementary material for: Quantification of the Pirimicarb Resistance Allele Frequency in Pooled Cotton Aphid (Aphis gossypii Glover) Samples by TaqMan SNP Genotyping Assay
Source: PLoS One. 2014 Mar 10;9(3):e91104. doi: 10.1371/journal.pone.0091104 (PMC3948748; doi:10.1371/journal.pone.0091104)
Supplement: Table S5 — Five standard points used for the reduced prediction model. (DOC) [file pone.0091104.s005.doc]

**Table S5.** Five standard points used for the reduced prediction model

| **RAF** | **Run1 T/S´** | **Run2 A/S** | **Run3 A/S** | **Run4 T/S** | **Run5 MP/S** | **Run6 MP/S** | **Run7 MP/S** |
| --- | --- | --- | --- | --- | --- | --- | --- |
|  | **k** | **k´** | **k´** | **k´** | **k´** | **k´** | **k´** |
| **100** | 0.924 | 0.908 | 0.917 | 0.916 | 0.917 | 0.888 | 0.925 |
| **80** | 0.791 | 0.752 | 0.755 | 0.802 | 0.821 | 0.826 | 0.849 |
| **50** | 0.588 | 0.564 | 0.559 | 0.615 | 0.665 | 0.687 | 0.705 |
| **20** | 0.392 | 0.359 | 0.347 | 0.444 | 0.453 | 0.446 | 0.508 |
| **0** | 0.255 | 0.246 | 0.233 | 0.248 | 0.184 | 0.165 | 0.161 |

**RAF**: Predefined Resistance allele frequency (RAF) expressed as percentage.

**k´** : Average of transformed fluorescence ratio from 3 replicates.
