# Supplementary material for: Quantification of the Pirimicarb Resistance Allele Frequency in Pooled Cotton Aphid (Aphis gossypii Glover) Samples by TaqMan SNP Genotyping Assay
Source: PLoS One. 2014 Mar 10;9(3):e91104. doi: 10.1371/journal.pone.0091104 (PMC3948748; doi:10.1371/journal.pone.0091104)
Supplement: Table S6 — Resistance allele frequencies (RAF) predicted from full and reduced prediction models in plasmid mix runs. (DOC) [file pone.0091104.s006.doc]

**Table S6.** Resistance allele frequencies (RAF) predicted from full and reduced prediction models in plasmid mix runs

| **RAF** | **Run1 T/S** | | | **Run2 A/S** | | | **Run3 A/S** | | | **Run4 T/S** | | |
| --- | --- | --- | --- | --- | --- | --- | --- | --- | --- | --- | --- | --- |
|  | ***k'*** | **Predicted RAF** | | ***k'*** | **Predicted RAF** | | ***k'*** | **Predicted RAF** | | ***k'*** | **Predicted RAF** | |
|  |  | **f-model** | **r-model** |  | **f-model** | **r-model** |  | **f-model** | **r-model** |  | **f-model** | **r-model** |
| **100** | **0.924** | **100.2** | **99.9** | **0.908** | **100.69** | **100.3** | **0.917** | **100.7** | **100.3** | **0.916** | **98.5** | **99.4** |
| **95** | 0.887 | 94.9 | 94.5 | 0.862 | 94.57 | 94.2 | 0.869 | 94.6 | 94.1 | 0.89 | 95.0 | 95.6 |
| **90** | 0.851 | 89.6 | 89.2 | 0.823 | 89.29 | 88.9 | 0.828 | 89.3 | 88.8 | 0.861 | 90.8 | 91.1 |
| **80** | **0.791** | **80.8** | **80.2** | **0.752** | **79.46** | **79.0** | **0.755** | **79.6** | **79.0** | **0.802** | **81.6** | **81.3** |
| **70** | 0.71 | 68.7 | 68.1 | 0.683 | 69.62 | 69.1 | 0.679 | 69.2 | 68.5 | 0.738 | 70.8 | 70.2 |
| **60** | 0.654 | 60.2 | 59.7 | 0.622 | 60.67 | 60.1 | 0.621 | 61.0 | 60.3 | 0.672 | 59.2 | 58.4 |
| **50** | **0.588** | **50.2** | **49.7** | **0.564** | **51.96** | **51.3** | **0.559** | **52.0** | **51.3** | **0.615** | **49.1** | **48.3** |
| **40** | 0.531 | 41.5 | 41.1 | 0.498 | 41.78 | 41.1 | 0.491 | 41.8 | 41.2 | 0.554 | 38.5 | 38.0 |
| **30** | 0.444 | 28.3 | 28.0 | 0.395 | 25.33 | 24.8 | 0.386 | 25.4 | 25.1 | 0.496 | 29.0 | 28.8 |
| **20** | **0.392** | **20.5** | **20.2** | **0.359** | **19.41** | **19.0** | **0.347** | **19.1** | **18.9** | **0.444** | **21.2** | **21.3** |
| **10** | 0.321 | 9.9 | 9.7 | 0.308 | 10.87 | 10.7 | 0.295 | 10.6 | 10.6 | 0.382 | 12.9 | 13.2 |
| **5** | 0.286 | 4.8 | 4.5 | 0.28 | 6.10 | 6.0 | 0.272 | 6.7 | 6.9 | 0.31 | 4.6 | 5.3 |
| **0** | **0.255** | **0.3** | **-0.1** | **0.246** | **0.24** | **0.4** | **0.233** | **0.1** | **0.4** | **0.248** | **-1.3** | **-0.4** |
|  |  |  |  |  |  |  |  |  |  |  |  |  |
| **R^2^** |  | 0.9995 | 0.9995 |  | 0.9978 | 0.9977 |  | 0.9976 | 0.9976 |  | 0.9985 | 0.9982 |

RAF = a / (1.0 + exp-(k'-b)/c)) + y_0_

| **a** |  | -428.2 | -602.1 |  | -305302.0 | 645.2 |  | -393350.0 | -11660.6 |  | -163.2 | -171.6 |
| --- | --- | --- | --- | --- | --- | --- | --- | --- | --- | --- | --- | --- |
| **b** |  | 0.5683 | 0.5854 |  | -15.5168 | -0.0798 |  | -14.8618 | -7.7762 |  | 0.6615 | 0.6934 |
| **c** |  | -0.7034 | -0.9978 |  | -2.3889 | 0.9425 |  | -2.1679 | -2.4357 |  | -0.2288 | -0.2398 |
| **y_0_** |  | 261.315 | 350.3871 |  | 415.7030 | -377.4233 |  | 372.0351 | 419.9332 |  | 138.8981 | 148.0560 |

f-model: RAF predicted based on full 13-standard-points

r-model: RAF predicted based on reduced 5-standard-points standard allele frequency 100, 0.8, 0.5, 0.2, 0.0 in bold) .

R^2^: coefficient of determination between predefined RAF and transformed fluorescence ratio with linear regression
