# Supplementary material for: Quantification of the Pirimicarb Resistance Allele Frequency in Pooled Cotton Aphid (Aphis gossypii Glover) Samples by TaqMan SNP Genotyping Assay
Source: PLoS One. 2014 Mar 10;9(3):e91104. doi: 10.1371/journal.pone.0091104 (PMC3948748; doi:10.1371/journal.pone.0091104)
Supplement: Table S7 — Resistance allele frequencies (RAF) predicted from full and reduced prediction models in aphid mix runs. (DOC) [file pone.0091104.s007.doc]

**Table S7.** Resistance allele frequencies (RAF) predicted from full and reduced prediction models in aphid mix runs

| **RAF** | **Run5 MP/S** | | | **Run6 MP/S** | | | **Run7 MP/S** | | |
| --- | --- | --- | --- | --- | --- | --- | --- | --- | --- |
|  | ***k'*** | **Predicted RAF.** | | ***k'*** | **Predicted RAF.** | | ***k'*** | **Predicted RAF** | |
|  |  | **f-model** | **r-model** |  | **f-model** | **r-model** | ***k’*** | **f-model** | **r-model** |
| **100** | **0.917** | **98.4** | **100.0** | **0.888** | **98.8** | **99.1** | **0.925** | **99.4** | **99.7** |
| **95** | 0.886 | 93.7 | 93.5 | 0.871 | 94.2 | 93.9 | 0.916 | 96.7 | 97.4 |
| **90** | 0.881 | 92.9 | 92.5 | 0.862 | 91.8 | 91.2 | 0.885 | 87.9 | 89.6 |
| **80** | **0.821** | **82.2** | **80.0** | **0.826** | **82.4** | **81.1** | **0.849** | **78.6** | **80.7** |
| **70** | 0.73 | 63.7 | 61.9 | 0.757 | 65.6 | 64.4 | 0.819 | 71.5 | 73.5 |
| **60** | 0.721 | 61.8 | 60.2 | 0.738 | 61.3 | 60.3 | 0.778 | 62.6 | 64.2 |
| **50** | **0.665** | **50.1** | **50.0** | **0.687** | **50.7** | **50.4** | **0.705** | **48.9** | **49.3** |
| **40** | 0.632 | 43.6 | 44.4 | 0.632 | 40.5 | 41.2 | 0.651 | 40.3 | 39.7 |
| **30** | 0.551 | 29.3 | 32.1 | 0.564 | 30.0 | 31.6 | 0.528 | 24.8 | 22.6 |
| **20** | **0.453** | **16.4** | **20.0** | **0.446** | **16.4** | **18.7** | **0.508** | **22.7** | **20.4** |
| **10** | 0.424 | 13.6 | 17.0 | 0.421 | 14.2 | 16.4 | 0.383 | 11.8 | 9.7 |
| **5** | 0.262 | 3.4 | 4.1 | 0.254 | 3.8 | 4.9 | 0.281 | 5.3 | 4.1 |
| **0** | **0.184** | **1.0** | **0.0** | **0.165** | **0.5** | **0.7** | **0.161** | **-0.6** | **-0.1** |
| R^2^ |  | 0.993 | 0.991 |  | 0.996 | 0.995 |  | 0.996 | 0.995 |

RAF = a / (1.0 + exp-(k'-b)/c)) + y_0_

| **a** |  | 130.2 | -211.8 |  | 246.9 | 47064106.4 |  | 1932038.1 | 228.8 |
| --- | --- | --- | --- | --- | --- | --- | --- | --- | --- |
| **b** |  | 0.7234 | 0.8856 |  | 0.9544 | 5.5325 |  | 4.6584 | 0.9577 |
| **c** |  | 0.1545 | -0.2531 |  | 0.2224 | 0.3591 |  | 0.3840 | 0.2224 |
| y_0_ |  | -2.8396 | 199.3470 |  | -6.4006 | -14.5043 |  | -16.3956 | -6.3087 |

f-model: RAF predicted based on full 13-standard-points

r-model: RAF predicted based on reduced 5-standard-points standard allele frequency 100, 0.8, 0.5, 0.2, 0.0 in bold) .

R^2^: coefficient of determination between predefined RAF and transformed fluorescence ratio with linear regression
