# Supplementary material for: Quantification of the Pirimicarb Resistance Allele Frequency in Pooled Cotton Aphid (Aphis gossypii Glover) Samples by TaqMan SNP Genotyping Assay
Source: PLoS One. 2014 Mar 10;9(3):e91104. doi: 10.1371/journal.pone.0091104 (PMC3948748; doi:10.1371/journal.pone.0091104)
Supplement: Table S8 — Transformed fluorescence ratio k’ between runs can be normalized to Run1 T/S by using five standards based on sigmoid function. (DOC) [file pone.0091104.s008.doc]

**Table S8.** Transformed fluorescence ratio k´ between runs can be normalized to Run1 T/S by using five standards based on sigmoid function.

| **Standard** | **Run1 T/S** | **Run2 A/S** | **Run3 A/S** | **Run4 T/S** | **Run5 MP/S** | **Run6 MP/S** | **Run7 MP/S** |
| --- | --- | --- | --- | --- | --- | --- | --- |
|  | **k'** | **k'** | **k'** | **k'** | **k'** | **k'** | **k'** |
| **100** | 0.924 | 0.908 | 0.917 | 0.916 | 0.917 | 0.888 | 0.925 |
| **80** | 0.791 | 0.752 | 0.755 | 0.802 | 0.821 | 0.826 | 0.849 |
| **50** | 0.588 | 0.564 | 0.559 | 0.615 | 0.665 | 0.687 | 0.705 |
| **20** | 0.392 | 0.359 | 0.347 | 0.444 | 0.453 | 0.446 | 0.508 |
| **0** | 0.255 | 0.246 | 0.233 | 0.248 | 0.184 | 0.165 | 0.161 |
| **y= a / 1.0 + exp-x -b)/c)) + Offset** | | | | | | | |
| **a** |  | -68.82 | 164.92 | -1.19 | 1.51 | 285709 | 1.65 |
| **b** |  | -7.77 | -10.04 | 0.7 | 0.91 | 5.5 | 0.99 |
| **c** |  | -2.63 | 2.55 | -0.25 | 0.27 | 0.36 | 0.23 |
| **offset** |  | 3.37 | -161.79 | 1.27 | 0.16 | 0.16 | 0.21 |
